# The virtual haptic back: A simulation for training in palpatory diagnosis

**DOI:** 10.1186/1472-6920-8-14

**Published:** 2008-04-03

**Authors:** John N Howell, Robert R Conatser, Robert L Williams, Janet M Burns, David C Eland

**Affiliations:** 1 Interdisciplinary Institute for Neuromusculoskeletal Research and the Department of Biomedical Sciences, Ohio University College of Osteopathic Medicine, Athens, OH 45701, USA; 2 Interdisciplinary Institute for Neuromusculoskeletal Research, and the Department of Mechanical Engineering, Russ College of Engineering, Ohio University, Athens, OH 45701, USA; 3 Interdisciplinary Institute for Neuromusculoskeletal Research, and the Department of Family Medicine, Ohio University College of Osteopathic Medicine, Athens, OH 45701, USA

## Abstract

**Background:**

Models and simulations are finding increased roles in medical education. The Virtual Haptic Back (VHB) is a virtual reality simulation of the mechanical properties of the human back designed as an aid to teaching clinical palpatory diagnosis.

**Methods:**

Eighty-nine first year medical students of the Ohio University College of Osteopathic Medicine carried out six, 15-minute practice sessions with the VHB, plus tests before and after the sessions in order to monitor progress in identifying regions of simulated abnormal tissue compliance. Students palpated with two digits, fingers or thumbs, by placing them in gimbaled thimbles at the ends of PHANToM 3.0^® ^haptic interface arms. The interface simulated the contours and compliance of the back surface by the action of electric motors. The motors limited the compression of the virtual tissues induced by the palpating fingers, by generating counterforces. Users could see the position of their fingers with respect to the back on a video monitor just behind the plane of the haptic back. The abnormal region varied randomly among 12 locations between trials. During the practice sessions student users received immediate feedback following each trial, indicating either a correct choice or the actual location of the abnormality if an incorrect choice had been made. This allowed the user to feel the actual abnormality before going on to the next trial. Changes in accuracy, speed and Weber fraction across practice sessions were analyzed using a repeated measures analysis of variance.

**Results:**

Students improved in accuracy and speed of diagnosis with practice. The smallest difference in simulated tissue compliance users were able to detect improved from 28% (SD = 9.5%) to 14% (SD = 4.4%) during the practice sessions while average detection time decreased from 39 (SD = 19.8) to 17 (SD = 11.7) seconds. When asked in anonymous evaluation questionnaires if they judged the VHB practice to be helpful to them in the clinical palpation and manual medicine laboratory, 41% said yes, 51% said maybe, and 8% said no.

**Conclusion:**

The VHB has potential value as a teaching aid for students in the initial phases of learning palpatory diagnosis.

## Background

Palpatory diagnosis plays an important role in medicine. It is through palpation that lymph nodes that are swollen and muscles in spasm, as well as neoplasms in breasts, prostate glands, testes and abdomens, are detected. Palpation is quick and inexpensive, but it is also subjective. In osteopathic medicine, as well as other disciplines oriented toward the musculoskeletal system, such as chiropractic, physical therapy and massage, students receive extensive training in palpating muscles, bones, joints and connective tissues in order to diagnose altered functional states that can be treated by manual methods. Training is typically done in laboratory settings in which students work on each other with teacher-student ratios that make it difficult for students to get the level of feedback they desire as to whether they are feeling what they are supposed to be feeling. These settings also seldom provide the range of ages and conditions typical of patient populations the students will eventually be treating.

The development of the virtual haptic back (VHB) was undertaken to address these limitations. It is a simulation of the contours and the tissue textures of human backs and is presented to users both haptically and graphically, i.e., by feel and by sight. The simulation is based on measurements of real backs, the contours being captured by 3D photography and the tissue texture being measured as tissue compliance (the inverse of stiffness) with a PHANToM 3.0 haptic interface (SensAble Technologies, Woburn, MA) used as a force-displacement probe. A pilot study with 21 osteopathic medical student volunteer subjects demonstrated that, with practice on the VHB, subjects improved their ability to detect regions of altered compliance on the VHB [1,2]. On a pretest they were, on average, only able to detect regions that differed in compliance by at least 40%; following eight practice sessions of the VHB they were able to detect regions that differed in compliance by as little as 11%. Anonymous evaluations provided by the student users indicated that they thought the practice sessions were very helpful to them in their clinical labs, where they were learning to palpate regions of altered tissue texture on their fellow students. The simulation provides immediate feedback to users as to the correctness of their diagnosis, something students felt was not optimally provided in the students labs. Based on these results and the recommendations of the osteopathic manipulative medicine teaching staff the VHB was incorporated into the curriculum for the fall of 2006.

The present results on a larger number of participants permitted comparison with earlier studies of the patterns of haptic discrimination and exploration [3-5].

## Methods

### Participants

Of the 112 first-year osteopathic medical students who began the study, 93 completed the pre- and post-tests and the six practice sessions in the required two week period. The results of four students were excluded because of failure to record the data from one of their six practice sessions, leaving an N of 89 for the haptic data. Of the 89, 18 failed to fill out the evaluation form following the sessions, leaving an N of 71 for the evaluation data. Because the evaluation forms were done entirely anonymously, we have no way of knowing which of the 89 filled out the forms. Although the VHB project was ruled exempt by the Institution Review Board of Ohio University and the students were required to do the VHB exercise for classroom purposes, we obtained signed consent forms which permitted us to use the results generated by individual students for research purposes.

### The Haptic Model

The model is based on measurements of the back of a 51 year old female in good health. Contours of the back were determined with a 3-D camera (Inspeck, 3-D Megacapturor II); tissue compliances were measured with a PHANToM 3.0 haptic interface fitted with a finger-sized probe through which force is applied stepwise while displacements were recorded [6]. For simulation of abnormalities of tissue texture, 12 rectangular regions of the back (3.5 × 4.0 cm) over the paraspinal musculature of the thorax, were programmed to exhibit compliances different from the normal regions above and below. These varied randomly in location, with respect to side and vertebral level, between trials. The severity of the abnormality was also varied in accordance with the protocol being used. The model permits palpation of individual spinous processes of the vertebra for orientation. Also for orientation, a graphic image of the back appeared on a monitor just behind the plane of the haptic back. The locations of the users' palpating fingers with respect to the haptic back were indicated by two color coded dots on the screen. For more details of the haptic back program, see [1].

### Hardware and software

The hardware consisted of two PHANToM 3.0 haptic interfaces (SensAble Technologies, Woburn, MA) programmed using C++, OpenHaptics Software Toolkit, GHOST^®^SDK (SensAble Technologies, Woburn, MA), and OpenGL for graphics. Electric motors of the PHANToM provided force feedback reflecting the contours and tissue textures of the back. Tissue texture variations were represented as altered compliances (inverse of stiffness) in response to compression of the back surface by the palpating fingers.

### The learning task

By palpation through the haptic interfaces, students located regions of abnormal tissue texture, i.e., regions of reduced compliance (increased stiffness). To register their localizations of the abnormal region they pressed a foot switch while holding a finger on the area they detected as abnormal. In the pre- and post-tests users localized abnormalities at 5 different levels of difficulty, with two trials at each level, starting with the easiest. In each practice session, students started at the easiest level. After two trials at each level, students could change to a different level, moving either up one level of difficulty or down. Eleven levels of difficulty were used in the practice sessions covering a wider range of difficulty than was utilized in the tests.

### Feedback during the learning task

While palpating the back the graphic image contained only the surface of the back. During the practice sessions, when students made an incorrect identification, a transparency function was activated that revealed the position of the vertebrae and ribs beneath the surface and outlined the region that was actually abnormal. Students could then feel the abnormal areas before going on to the next trial.

### The protocol

The protocol consisted of the following:

1. Signing of consent form and 5-min orientation to the VHB

2. Pre-test – 10 min.

3. Practice sessions – N = 6, 15 min. each over a 2 week period

4. Post-test – 10 min. and evaluation

Students were required to carry out the VHB practice sessions, but they were not required to permit us to use their data for research purposes. The consent form gave that permission. A research staff person oriented the student users to the use of the VHB. The pre-test was done immediately following the orientation, and the first practice session was typically also done in the same session. Laboratory keys were made available so the subsequent practices could be done at the users' convenience. No more than one practice session could be done on any given day. The post-test was typically done immediately after the last practice session. Upon completion users filled out anonymous evaluation forms.

### Method of performance evaluation

Levels of task difficulty were defined by the Weber fraction, i.e., the compliance difference between the abnormal region and the adjacent, normal region, divided by the compliance of the normal region. Multiplying by 100 gives the percentage difference in compliance between normal and abnormal regions. Levels of difficulty ranged from 44% (easy) to 4% (difficult) in the practice sessions and from 39% to 7% in the tests before and after practice. Mastery at any given difficulty level was defined as correct identification ≥55% of trials, approximately half way between the percentage expected by chance (≤8.3%) and 100%. Because there were 12 possible abnormal regions, the chance level of a correct identification cannot be more than 1 in 12 (8.3%). The 12 regions had little space in between them and covered most of the paraspinal area. Chance level could in principle be lower than 8.3%, however, since the user could have had his/her identifying finger placed lateral or medial to any of the abnormal regions when the footswitch was pressed.

## Results

### Palpatory performance

**Figure 1:**
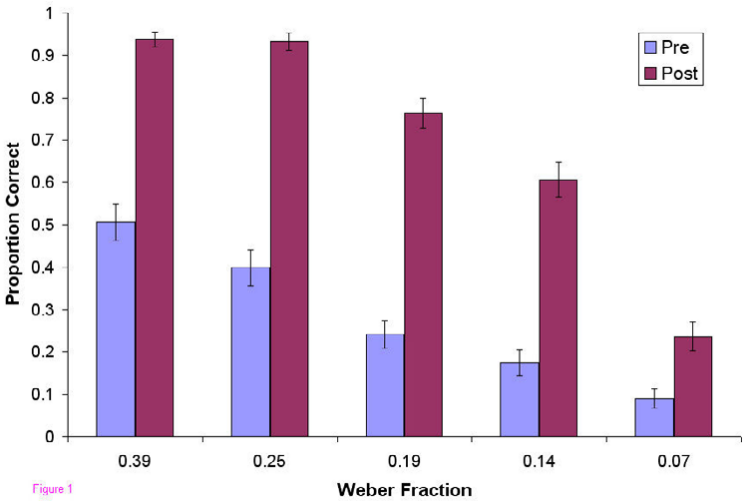
**Proportion Correct vs. Weber Fraction**. Proportion of correct responses in the pre- and post-tests as a function of difficulty level.

**Figure 2:**
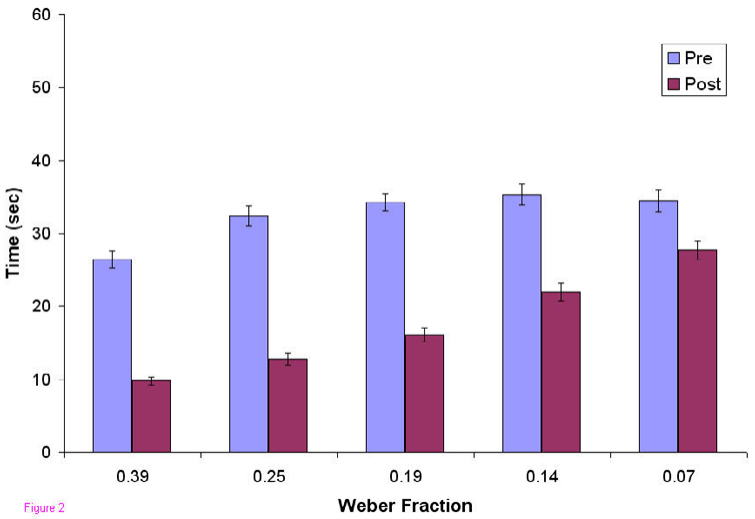
**Response Time vs. Weber Fraction**. Response times in pre- and post-tests as a function of difficulty level. Maximum time permitted for each localization was 60 sec.

**Figure 3:**
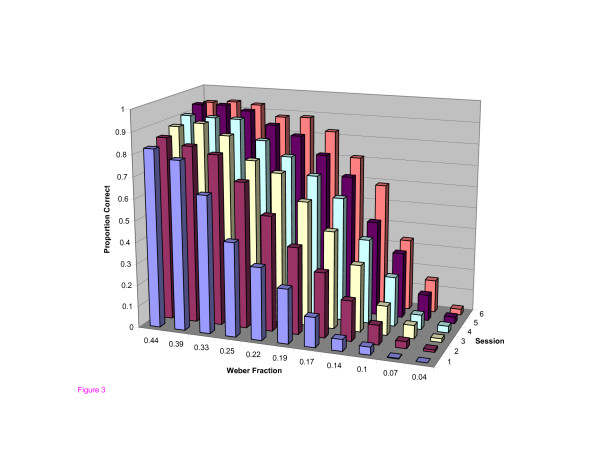
**3D plot of Proportion correct vs. Practice Session vs. Weber Fraction**. Proportion of correct responses in the practice sessions plotted against task difficulty and practice session number.

In the pre-test, users as a group, did not exhibit mastery at any of the five levels of difficulty tested (Figure 1). In the post-test, after the 6 practice sessions, mastery was achieved at all levels except the most difficult. Users also became faster at localizing the abnormalities (Figure 2). Improvement in accuracy during the successive practice sessions was greatest in the intermediate levels of difficulty and less at the extremes (Figure 3).

**Figure 4:**
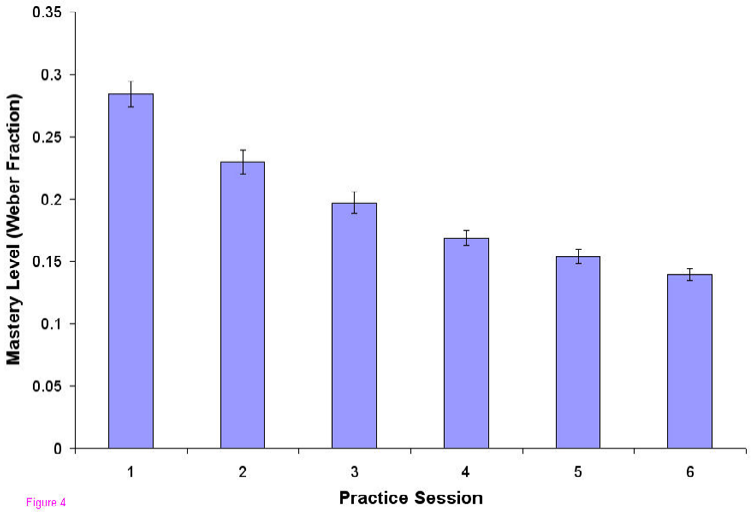
**Mastery Level vs. Practice Session**. Improvement in mastery level, indicated as the lowest Weber fraction at which at least 55% of the responses were correct, as a function of practice session number. All visits are significantly different (F = 111, P < 0.001, η^2 ^= 0.55).

For the group, the average mastery level improved from a Weber fraction of 0.28 to 0.14 over the six practice sessions (Figure 4), i.e, improvement from detection of only a 28% compliance difference to a 14% difference. The average time per localization fell from 39 sec to 17 sec, but the average force exerted by the users did not change significantly over the sessions (range 2.3 to 2.5 N) (data not shown).

**Figure 5:**
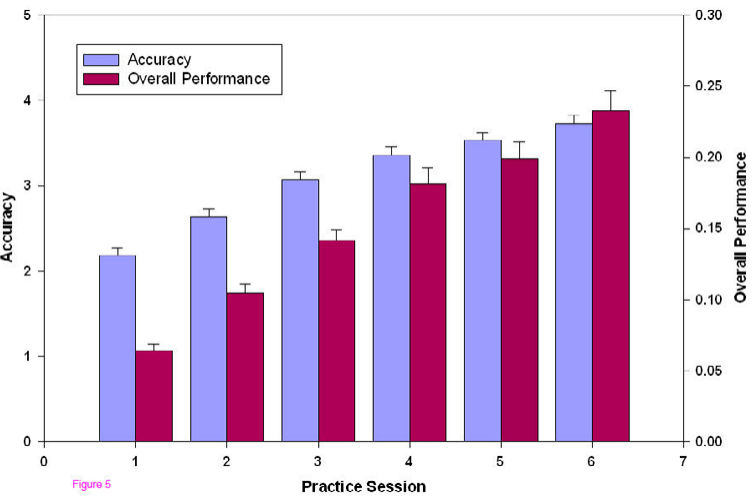
**Accuracy and overall performance vs. Practice Session**. Accuracy and overall performance, plotted against the practice session number. Accuracy (blue) is defined as the proportion correct divided by the Weber fraction. All visits are significantly different (F = 96, P < 0.001, η^2 ^= 0.52). Overall performance (red) includes both accuracy and speed and is defined as the proportion correct divided by the product, (Weber fraction)*(time). All visits are significantly different (F = 87, P < 0.001, η^2 ^= 0.49).

An overall measure of accuracy, the proportion correct/Weber fraction, showed significant improvement between each session (Figure 5). An overall measure of performance that included both accuracy and speed, namely proportion correct/((Weber fraction)(time)), also showed continuous improvement throughout the sessions (Figure 5).

**Figure 6:**
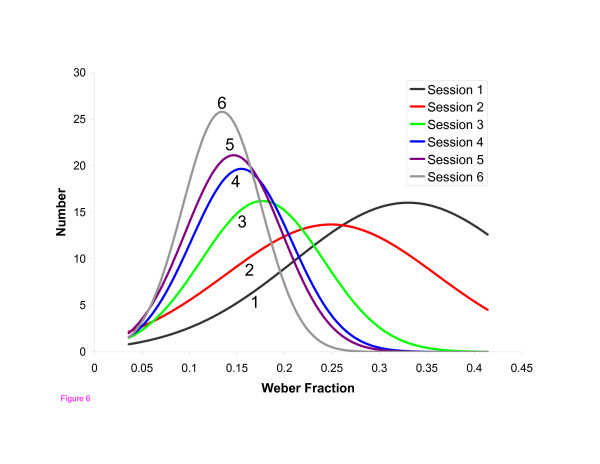
**Gaussian fit to mastery data**. The number of student users plotted against the Weber fraction of the difficulty level at which mastery was demonstrated. The data for each practice session have been fitted with Gaussian distributions in order to see more easily the shift toward lower Weber fractions with each session. The R^2 ^values (goodness of fit) for sessions 1 through 6 were 0.87, 0.67, 0.77, 0.81, 0.93, and 0.92, respectively.

The improvement in group performance can also be seen in plots of the number of students achieving mastery at various difficulty levels at each successive practice session. (Figure 6). As students mastered the palpatory process, the class mean improved and the distribution became tighter.

### Participant evaluation

These results are not surprising. Improvement in performance comes with practice in any task. The important question is if this virtual world practice has any carry over into palpation in a clinical setting. At the time these first year medical student users were participating in this study, they were also taking a laboratory course in manipulative medicine, a course that begins with training in palpation. At the conclusion of their sessions with the VHB, they filled out evaluation forms inviting their comments and containing three specific questions. 71 of the 89 participants returned the forms. Two questions asked if the students thought that practice on the VHB would be helpful to the development of their palpatory skills (Table 1). 41% indicated that they thought the experience they had with the VHB would be useful in the development of their palpatory skills; 8% thought that it would not be helpful. Only 17% were convinced that further practice would be of additional help. The third question asked them to rate the realism of the simulation on a 0 to 10 scale; the result was a mean of 5.3 with a standard deviation of 2.0.

**Table 1:** Post Test Questionnaire

Question	Yes	Maybe	No
Do you think this practice with the haptic back will be of help to you in the development of your palpatory skills in the OMM lab?	29	36	6
Do you think further practice with the haptic back would be of help to you in the development of your palpatory skills?	12	39	20

## Discussion

### Comparison of results to preceding study

The results presented here confirm and extend the results of a pilot study with 21 first- year, medical student volunteers in the fall of 2005 [1,2], showing that students improve with practice and that they judge the practice to be helpful in the development of their palpatory skills. The results with the volunteers showed higher palpatory achievement, reaching a Weber fraction of 9% with 8 practice sessions, compared to 14% achieved in this study with 6 sessions. Subjects in the pilot study also rated the experience as more helpful to their palpatory skill development, with 81% "yes" answers to question #1 in 1, compared to only 33% in this study. The biggest difference in the two studies was that the VHB practice was a required exercise in the Manipulative Medicine course in this study. Some students appeared to resent being required to participate and probably did not give their full efforts to achieve mastery.

Another difference in the two studies was that the task was somewhat more difficult in this study. The model used in the previous study was a back created by the programmer to have uniform compliance throughout, except at the region of abnormality. The model used for the current study was based on the contours and measured compliance of the back of a 51 year old female. The background compliance was not uniform, requiring the users to detect the abnormality in compliance against a non-uniform background.

A final difference was that, based on the pilot study, which seemed to indicate that most of the learning was complete in 6 sessions, we reduced the number of practice sessions from 8 to 6. Figures 4, 6 and 8 suggest that improvement was still occurring between the last two practice sessions in the current study, although it appeared to be approaching an asymptote.

The results from the first two evaluation questions in both studies suggested that the VHB, in its current form, is most helpful early in clinical palpatory training, when students are just beginning to learn to trust information coming from their palpating fingers. The VHB provides immediate feedback as to whether the student identification of the abnormal area is correct. Students appreciated that, because they often claim that they do not get enough feedback in the clinical lab to know if what they are feeling on each other is correct.

In the current study students rated the realism of the simulation at 5.3, compared to 6.5 in the pilot study, despite the fact that the back model was clearly more realistic in this study, being based on measurements of a real back. The lower rating may reflect attitudes resulting from being required to participate.

### What is the limit of palpatory discrimination of compliance that can be detected?

Using a haptic device (PHANToM 1.5) similar to the ones used in this study and comparable compliance values, DeGersem [7] and De Gersem et al. [3] studied 5 subjects and, reported they could detect compliance differences in the range of 8 to 12%, somewhat better than the mean of 14% achieved in our study. Ten of our 89 student subjects were, however, able to demonstrate mastery at 7%. The task was simpler in the De Gersem study, determining only which of two haptic surfaces is stiffer, but participants in that study did not have the opportunity to practice as did the subjects in our study. It is not yet clear how much, if any, improvement would be brought about by further practice. The declining rate of improvement with successive practice sessions suggests that a limit was being approached.

### How do subjects assess the two components of compliance, namely force and displacement?

In principle, a person sensing compliance can either apply a (subjectively) known force and assess the displacement, or apply a (subjectively) known displacement and assess the force level required. The approach most commonly used by our subjects to find the abnormal area on the VHB was to run two fingers simultaneously up and down the paravertebral region exerting fairly constant force and searching for the bump that reflected the abnormally stiff region. The forces applied remained relatively constant throughout the trials, 2.30 to 2.52 N. This is consistent with the results of Walker and Tan [4] who showed, using a PHANToM 1.5 for compliance detection, that forces tended to stay constant while subjects were feeling haptic surfaces, apparently registering their sense of which surface was more compliant by the displacement achieved. In a surface height discrimination using surface compliance comparable to ours, their subjects could detect a 0.56 mm height difference. Assuming that our subjects applied constant force, the displacement they detected in discriminating a 14% compliance difference, given an average background compliance of 2.52 mm/N, was 0.85 mm. Twenty-eight subjects achieved a Weber fraction of 10% and 10 subjects achieved a Weber fraction of 7%, corresponding to sensing displacements of 0.6 mm and 0.44 mm respectively.

### Time required for palpation

Subjects in our study were often able to locate obvious abnormalities with a single pass down the back simultaneously with 2 fingers (or thumbs), stopping their search at the site of the abnormality. At the easiest level 19 students found the abnormality within 5 seconds. Lederman and Klatzky [5] reported minimum times of 400 to 600 msec for subjects to identify by a single finger touch whether a surface was hard rubber or soft (foam) rubber (compliance ratios of more than 20 fold), but no searching for location was involved, and there was no subtlety in compliance differences. In more difficult discriminations, done not with compliance differences, but with relative surface smoothness, their subjects took between longer, 962 and 1220 msec in two reported tasks. Purdy et al. [8] examined more extensively the relation between localizing and identifying a haptic feature, and likewise revealed a cost in searching time associated with processing location information. In our study subjects also took longer as the compliance differences became more subtle as shown in the pre- and post-test data in fig. 2, taking on average 10 sec and 28 sec respectively on the easiest and hardest discriminations on the post-test.

### Patterns of palpation

Students were able to palpate with two fingers of one hand or one finger from each hand. They could also use two thumbs or a thumb and a finger. Some students experimented with different options. No quantitative evaluation of these patterns was carried out. A common pattern at the more difficult levels was for subjects to switch from palpating simultaneously with two fingers to using one at a time, as if the added noise associated with two simultaneous inputs swamped out the subtle difference detected by one finger. Lederman and Klatzky [5] examined the increased time it took for their subjects to detect the presence of a haptically different surface as more fingers became involved in the detection process when the discrimination task was difficult. They characterized this as a "switch to a serial search," which may be analogous to our subjects' shifting to a one finger search.

### Validation

Does improved performance on the VHB translate into better palpatory diagnosis of real patients? The survey results, from student users in this study and in the preceding pilot study, suggest that it does and constitute a degree of face validation. The student users for the most part indicated that practice with the simulation was helping them in the laboratory. An objective demonstration of improvement in palpatory performance would provide firmer evidence. In the absence of independent objective measures of palpatory performance, however, this is hard to achieve. There is currently no objective standard of palpatory skill against which to measure performance improvement that might result from practice with the VHB.

One might also expect that individuals experienced in palpatory diagnosis might do demonstrably better at the VHB than first year osteopathic medical students. Pilot efforts tell us, however, that this is not the case. This does not necessarily indicate that improvement on the VHB for students has no value in terms of transfer of skill to the real world. In our experience, physicians have more difficulty in getting oriented to the simulation than beginning students, perhaps because of the absence of cues to which they are accustomed. In an effort to provide at least some of these cues, current work is directed toward placing the palpating fingers (in the VHB probes) within a cutout area of a mannequin. Programming efforts are also underway to permit the VHB user to grasp a virtual shoulder or elbow to produce passive side bending or rotation in the virtual patient, while sensing the compliance changes in the back associated with this input of gross motion. Much more work is required to measure compliance changes in patients with somatic dysfunction objectively and to include in the simulation the more complex patterns of tissue texture change that have been described clinically [9].

## Conclusion

Six training sessions on the VHB improved palpatory speed and accuracy of first-year osteopathic medical students required to do the sessions. As a group, prior to training, students were unable to detect by palpation compliance differences on the VHB of 39% or less; after training they were able to detect differences as little as 14%. 41% of the student users indicated that they thought the experience was useful to them in the development of their palpatory skills, which were being taught in a concurrent clinical course. Only 8% of the student judged that it would not be helpful to them. These results indicate that the VHB has potential as a useful teaching aid for students learning palpatory diagnosis.

## Abbreviations

VHB – Virtual Haptic Back

## Competing interests

The author(s) declare that they have no competing interests.

## Authors' contributions

JNH provides the overall supervision of the project, and is the principle author of the grant that funded the project, and author of first draft of this manuscript. RRC is the principal programmer of the Virtual Haptic Back, principal data analyst, participated in the experimental design, and handled day-to-day participation of subjects in the study. RLW supervised the engineering aspects of the project, and participated in the experimental design and interpretation of data.

DCE and JMB supervised the medical education aspects of the project, participated in the experimental design and interpretation of data, and are instructors of the Manipulative Medicine course in the context of which the study was carried out. All authors have read and approved the final manuscript.

## Pre-publication history

The pre-publication history for this paper can be accessed here:


